# Meta‐analysis of prognostic factors for patients with colorectal peritoneal metastasis undergoing cytoreductive surgery and heated intraperitoneal chemotherapy

**DOI:** 10.1002/bjs5.50179

**Published:** 2019-06-27

**Authors:** S. Hallam, R. Tyler, M. Price, A. Beggs, H. Youssef

**Affiliations:** ^1^ Institute of Cancer and Genomic Sciences University of Birmingham Birmingham UK; ^2^ Institute of Applied Health Research University of Birmingham Birmingham UK; ^3^ Colorectal Surgery, Good Hope Hospital University Hospitals Birmingham NHS Foundation Trust Birmingham UK

## Abstract

**Background:**

Up to 15 per cent of colorectal cancers present with peritoneal metastases (CPM). Cytoreductive surgery and heated intraperitoneal chemotherapy (CRS + HIPEC) aims to achieve macroscopic tumour resection combined with HIPEC to destroy microscopic disease. CRS + HIPEC is a major operation with significant morbidity and effects on quality of life (QoL). Improving patient selection is crucial to maximize patient outcomes while minimizing morbidity and mortality. The aim of this study was to identify prognostic factors for patients with CPM undergoing CRS + HIPEC.

**Methods:**

A systematic search of MEDLINE, Embase and Cochrane Library electronic databases was performed using terms for colorectal cancer, peritoneal metastasis and CRS + HIPEC. Included studies focused on the impact of prognostic factors on overall survival following CRS + HIPEC in patients with CPM.

**Results:**

Twenty‐four studies described 3128 patients. Obstruction or perforation of the primary tumour (hazard ratio (HR) 2·91, 95 per cent c.i. 1·5 to 5·65), extent of peritoneal metastasis as described by the Peritoneal Carcinomatosis Index (PCI) (per increase of 1 PCI point: HR 1·07, 1·02 to 1·12) and the completeness of cytoreduction (CC score above zero: HR 1·75, 1·18 to 2·59) were associated with reduced overall survival after CRS + HIPEC.

**Conclusion:**

Primary tumour obstruction or perforation, PCI score and CC score are valuable prognostic factors in the selection of patients with CPM for CRS + HIPEC.

## Introduction

Colorectal peritoneal metastasis (CPM) occurs in up to 15 per cent of patients with colorectal cancer^1^, ^2^, ^3^. The prognosis of patients with CPM is poor: untreated median overall survival (OS) is just 6 months^4^. With systemic chemotherapy, median OS is improved to up to 20 months^5^, ^6^, ^7^. The standard for CPM is cytoreductive surgery and heated intraperitoneal chemotherapy (CRS + HIPEC), which may improve median OS by 20–63 months^8^, ^9^, ^10^, ^11^, ^12^, ^13^, ^14^, ^15^, ^16^.

CRS + HIPEC is a long, high‐cost operation associated with a protracted inpatient and high‐dependency or intensive care unit stay, and an associated mortality rate of 1–12 per cent and morbidity rate of 7–63 per cent^11^, ^14^, ^16^, ^17^, ^18^, ^19^, ^20^, ^21^, ^22^, ^23^. Improving patient selection is therefore crucial to maximize patient outcomes whilst minimizing morbidity and mortality.

Variation in outcomes for CRS + HIPEC can be explained in part by patient selection, for example necessitating the ability to achieve complete cytoreduction at CRS, the exclusion of patients with extensive CPM as assessed by Sugarbaker's Peritoneal Carcinomatosis Index (PCI), and selection of patients with minimal co‐morbidity and good performance status.

Several clinicopathological variables that impact on survival have been identified in the literature, including lymph node (LN) status, tumour differentiation and histological findings, the completeness of cytoreduction (CC score) and PCI. There is wide variation, however, in the variables reported by studies and selection criteria for centres performing CRS + HIPEC worldwide, and in turn in their outcomes.

Studies reporting prognostic factors for CRS + HIPEC are cohort in design with small samples, and each examines numerous and varying prognostic factors on differing scales of measurement. No consensus exists as to which prognostic factors contraindicate CRS + HIPEC, or predict a good outcome. A comprehensive evidence synthesis is therefore called for to determine relevant prognostic factors.

The aim of this systematic review and meta‐analysis was to analyse all prognostic factors affecting OS in patients with CPM undergoing CRS + HIPEC.

## Methods

A comprehensive literature search was conducted in accordance with the PRISMA guidelines^24^. MEDLINE, Embase and Cochrane Library electronic databases, registers of clinical trials (ClinicalTrials.gov and the WHO International Clinical Trials Registry) and the Conference Proceedings Citation Index, Zetoc, were searched from inception to the present. The search strategy captured terms for colorectal cancer, peritoneal metastasis and CRS + HIPEC techniques, separated by the Boolean operator ‘AND’. For an example search strategy, see *Appendix* S1 (supporting information). Searches were supplemented by a hand search of selected journals and the reference lists of all included studies.

### Study selection

English‐language articles were eligible for inclusion if they reported on the impact of prognostic factors on OS in patients with CPM undergoing CRS + HIPEC. Where multiple studies described the same cohort of patients, the largest and most complete data set was included. Review articles, case reports and case series of fewer than ten patients were excluded. Additional exclusion criteria included studies involving patients with a primary tumour other than colorectal cancer and studies in which a proportion of the cohort did not receive combined CRS + HIPEC.

After screening the titles and abstracts, articles fulfilling the eligibility criteria were identified and their full‐text publications reviewed. Literature search and study selection were done independently by two researchers, and any disagreements were resolved by discussion with senior reviewers. After qualitative assessment, articles were screened to ensure they presented adequate statistical information to be included in the meta‐analysis: hazard ratios (HRs) with confidence intervals or Kaplan–Meier curves with the number of events and patients at risk. When HRs, confidence intervals or *P* values were not provided directly, the methods of Tierney *et al*.^25^ were used to estimate them indirectly from Kaplan–Meier curves, when presented in adequate detail with the numbers of events and patients at risk^26^.

### Assessment of risk of bias

The quality and risk of bias of individual studies was assessed using the Quality in Prognosis Studies (QUIPS) tool^27^. This tool reviews each study according to six criteria: study participation, attrition, prognostic factor measurement, outcome measurement, confounding factors, and statistical analysis and reporting. Two authors scored all articles independently.

### Data extraction

Data were extracted independently by two reviewers using a dedicated and piloted data extraction form. The number of patients, study design, patient demographics, tumour characteristics, use of adjuvant and neoadjuvant regimens, CRS + HIPEC techniques, survival and prognostic factors were recorded.

The unadjusted HR and its 95 per cent c.i. and *P* value were extracted. When adjusted HRs (with confidence intervals and *P* values) were reported, these were extracted along with the set of adjustment factors used. If HRs, confidence intervals or *P* values were not provided directly, the methods of Tierney and colleagues^25^ were used to estimate them indirectly from Kaplan–Meier curves, when presented in adequate detail with the numbers of events and patients at risk^26^.

Prognostic factors reported on a continuous scale were extracted. If results were categorized into three or more categories, results for each comparison were extracted and, when clinically relevant, were grouped to form a binary comparison.

### Prognostic factor selection

All prognostic factors described adequately and reported by two or more independent studies were included.

### Statistical analysis

Owing to clinical and methodological heterogeneity, a random‐effects meta‐analysis was used (on the log(HR) scale) using the method of DerSimonian and Laird^28^. The combined effect size was described by the pooled HR, its confidence interval and *P* value, with *P* < 0·050 considered significant. For prognostic factors reported by more than two studies, a 95 per cent prediction interval (a measure of the variation in treatment effects) is presented^29^. Heterogeneity was also described by the *I* ^2^ statistic^30^. All analyses were performed in STATA® version 15 (StataCorp, College Station, Texas, USA).

## Results

**Figure 1:**
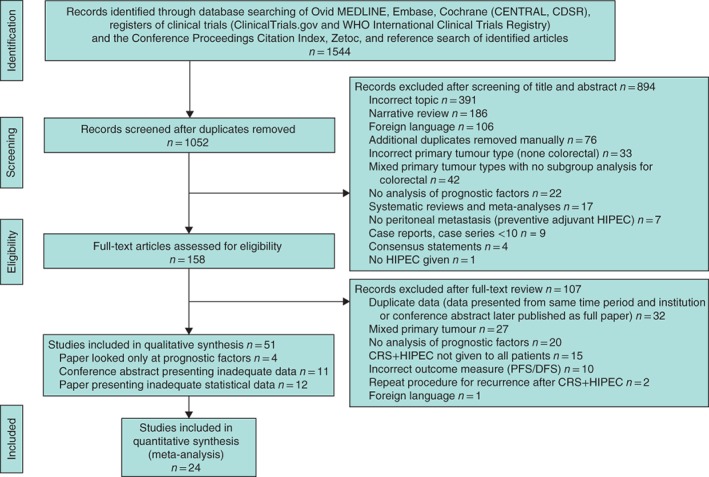
PRISMA diagram for the review CENTRAL, Cochrane Central Register of Controlled Trials; CDSR, Cochrane Database of Systematic Reviews; DARE, Database of Abstracts of Reviews of Effects; HIPEC, heated intraperitoneal chemotherapy; CRS, cytoreductive surgery; PFS, progression‐free survival; DFS, disease‐free survival.

The final literature search was performed on 3 April 2018. Literature searches (after removal of duplicates) identified 1052 records. Titles and abstract screening identified 158 full‐text articles for review. Of these, 51 studies met the inclusion criteria. Twenty‐four unique studies^11^, ^18^, ^20^, ^31^, ^32^, ^33^, ^34^, ^35^, ^36^, ^37^, ^38^, ^39^, ^40^, ^41^, ^42^, ^43^, ^44^, ^45^, ^46^, ^47^, ^48^, ^49^, ^50^, ^51^ reporting on 3128 patients with CPM presented adequate data to be included in the meta‐analysis (*Fig*. 1). Of the 24 cohort studies, six^18^, ^34^, ^37^, ^40^, ^44^, ^47^ were prospective and the remaining 18^11,20,31–33,35,36,38,39,41–43,45,46,48–51^ were retrospective (*Table* S1, supporting information).

A number of studies presented both an unadjusted and adjusted HR. As adjustment factors varied widely between studies (*Table* S2, supporting information), meta‐analysis was stratified according to whether the HR was unadjusted or adjusted.

### Quality of research

There was a low risk of bias from study participation. The moderate risk of bias due to study attrition reflects the poor reporting of loss to follow‐up. There was a low risk of bias due to prognostic factor measurement. Prognostic factors were objective, clearly defined and clinically relevant. Reporting of all prognostic factors and treatment variations was incomplete in the majority of studies, resulting in a moderate risk of bias. The presentation of results and analytical strategy was sufficient in the majority of studies resulting in a low risk of bias (*Table* S3, supporting information).

Pooled median OS across all studies was 32 (range 12·2–51) months, with a pooled median follow‐up of 28·1 (13·3–62·4) months (*Table* S4, supporting information).

### Patient factors

#### *Age*

**Figure 2:**
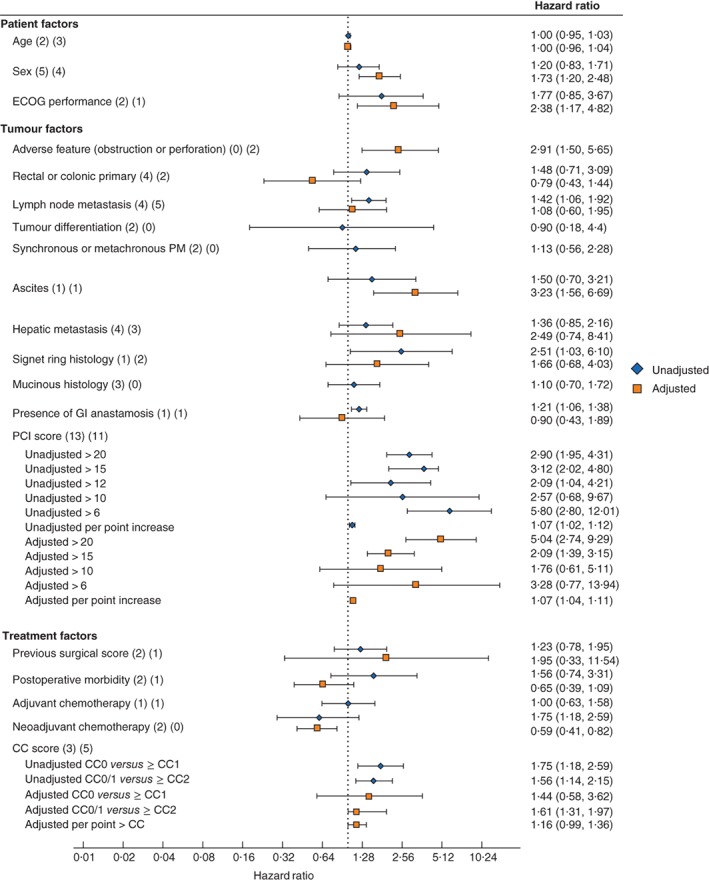
Effect of prognostic factors on overall survival Hazard ratios are shown with 95 per cent confidence intervals. Values in parentheses after each factor indicate the numbers of studies providing unadjusted and adjusted hazard ratios respectively. ECOG, Eastern Cooperative Oncology Group; PM, peritoneal metastasis; GI, gastrointestinal; PCI, Peritoneal Carcinomatosis Index; CC, completeness of cytoreduction.

The pooled median age of patients included was 54 (range 45·5–69·3) years. Eleven studies^18^, ^20^, ^33^, ^34^, ^37^, ^40^, ^42^, ^48^, ^49^, ^50^, ^52^ reported on age as a prognostic factor and ten presented adequate data to be included in the meta‐analysis. Five studies categorized age into binary outcomes that could not be combined meaningfully. The pooled unadjusted HR was 1·00 (95 per cent c.i. 0·98 to 1·03) (*I* ^2^ = 38·5 per cent) and adjusted HR was 1·00 (0·96 to 1·04) (*I* ^2^ = 69·3 per cent), for an increase in age of 1 year (*Fig*. 2; *Fig*. S1, supporting information). These data provide no evidence that age is a useful predictor of OS.

#### *Sex*

Nine studies^18^, ^20^, ^34^, ^36^, ^37^, ^40^, ^42^, ^51^, ^52^ reported on the effect of sex on OS and eight presented adequate data to be included in the meta‐analysis. The pooled unadjusted HR for male sex was 1·20 (95 per cent c.i. 0·83 to 1·71) (*I* ^2^ = 33·8 per cent) and adjusted HR was 1·73 (1·20 to 2·48) (*I* ^2^ = 11·6 per cent) (*Fig*. 2; *Fig*. S1, supporting information). It is therefore unclear whether sex is a useful predictor of OS.

#### *Eastern Cooperative Oncology Group performance status*

Five studies^18^, ^31^, ^43^, ^52^, ^53^ reported on the influence of Eastern Cooperative Oncology Group (ECOG) status on OS, and three presented adequate data to be included in the meta‐analysis. The pooled unadjusted HR for an ECOG score of at least 2 was 1·77 (95 per cent c.i. 0·85 to 3·67) (*I* ^2^ = 0 per cent) (*Fig*. 2; *Fig*. S1, supporting information). These data provide no evidence that ECOG is a useful predictor of OS.

### Tumour factors

#### *Adverse primary tumour features*

Three studies^18^, ^33^, ^52^ reported on the influence of adverse features of the primary tumour (obstruction or perforation) on OS; two presented adequate data to be included in the meta‐analysis. The pooled adjusted HR for adverse features of the primary tumour was 2·91 (95 per cent c.i. 1·5 to 5·64) (*I* ^2^ = 0 per cent) (*Fig*. 2; *Fig*. S1, supporting information). These data indicate that adverse primary features are a useful predictor of decreased OS (*Table* S5, supporting information).

#### *Rectal or colonic primary*

Eight studies^20^, ^34^, ^36^, ^40^, ^43^, ^47^, ^53^, ^54^ reported on the influence of a rectal or colonic primary on OS and six presented adequate data to be included in the meta‐analysis. The pooled unadjusted HR for rectal primary (compared with colonic primary) was 1·48 (95 per cent c.i. 0·71 to 3·09) (*I* ^2^ = 73·8 per cent) (*Fig*. 2; *Fig*. S1, supporting information). These data provide no evidence that a rectal primary is a useful predictor of OS.

#### *Lymph node metastasis*

Eleven studies^11^, ^18^, ^33^, ^34^, ^35^, ^37^, ^48^, ^49^, ^50^, ^53^, ^54^ reported on the effect of LN status on OS; nine presented adequate data to be included in the meta‐analysis. The pooled unadjusted HR for positive LNs (compared with negative LNs) was 1·42 (95 per cent c.i. 1·06 to 1·92) (*I* ^2^ = 0 per cent) and adjusted HR was 1·08 (0·60 to 1·95) (*I* ^2^ = 68·6 per cent) (*Fig*. 2; *Fig*. S1, supporting information). It is therefore unclear whether lymph nodes are a useful predictor of OS.

#### *Tumour differentiation*

Two studies^34^, ^40^ reported on the effect of primary tumour differentiation on OS. The pooled unadjusted HR for well differentiated tumours was 0·90 (95 per cent c.i. 0·18 to 4·40) (*I* ^2^ = 72·4 per cent) (*Fig*. 2; *Fig*. S1, supporting information). These data provide no evidence that primary tumour differentiation is a useful predictor of OS.

#### *Timing of colorectal peritoneal metastasis (synchronous or metachronous)*

Three studies^11^, ^40^, ^54^ reported on the effect of the timing of CPM (synchronous or metachronous) on OS. Two presented adequate data to be included in the meta‐analysis. The pooled unadjusted HR for synchronous CPM was 1·13 (95 per cent c.i. 0·56 to 2·28) (*I* ^2^ = 57·1 per cent) (*Fig*. 2; *Fig*. S1, supporting information), indicating that the timing of CPM is not a useful predictor of OS.

#### *Ascites*

Two studies^18^, ^40^ reported on the effect of malignant ascites on OS. One paper^40^ presented an unadjusted HR (1·50, 95 per cent c.i. 0·70 to 3·21) and one^18^ an adjusted HR (3·23, 1·56 to 6·69) (*Fig*. 2; *Fig*. S1, supporting information), so it was not possible to provide a pooled effect estimate.

#### *Hepatic metastasis*

Eleven studies^18^, ^31^, ^34^, ^35^, ^36^, ^37^, ^38^, ^43^, ^44^, ^47^, ^50^ reported on the effect of surgically treated hepatic metastasis on OS. Seven presented adequate data to be included in the meta‐analysis. The pooled unadjusted HR for surgically treated hepatic metastasis was 1·36 (95 per cent c.i. 0·85 to 2·16) (*I* ^2^ = 43·6 per cent) and the adjusted HR was 2·49 (0·74 to 8·41) (*I* ^2^ = 85·3 per cent) (*Fig*. 2; *Fig*. S1, supporting information). These data provide no evidence that the presence of surgically treated hepatic metastasis is a useful predictor of OS.

#### *Signet ring histology*

Three studies^11^, ^42^, ^46^ reported on the effect of signet ring histology on OS. Two included adequate data to be included in the meta‐analysis. The pooled adjusted HR for signet ring histology was 1·65 (95 per cent c.i. 0·68 to 4·03) (*I* ^2^ = 0 per cent) (*Fig*. 2; *Fig*. S1, supporting information), suggesting that signet ring histology is not a useful predictor of OS.

#### *Mucinous histology*

Four studies^11^, ^43^, ^46^, ^49^ reported on the influence of mucinous histology on OS. Three studies presented adequate data to be included in the meta‐analysis. The pooled unadjusted HR for mucinous histology was 1·10 (95 per‐cent c.i. 0·70 to 1·72) (*I* ^2^ = 0 per cent) (*Fig*. 2; *Fig*. S1, supporting information), suggesting that mucinous histology is not a useful predictor of OS.

#### *Gastrointestinal anastomosis in CRS + HIPEC*

Two studies^18^, ^43^ reported on the effect of one or more gastrointestinal anastomoses on OS. One paper^43^ presented an unadjusted HR (1·21, 95 per cent c.i. 1·06 to 1·38) and the other^18^ an adjusted HR (0·90, 0·43 to 1·89) (*Fig*. 2; *Fig*. S1, supporting information), so it was not possible to provide a pooled effect estimate.

#### *Peritoneal Carcinomatosis Index*

Eighteen studies^11^, ^20^, ^31^, ^32^, ^33^, ^34^, ^35^, ^36^, ^37^, ^38^, ^40^, ^41^, ^43^, ^45^, ^49^, ^50^, ^54^, ^55^ reported on the effect of the extent of CPM as described by the PCI; 16 studies presented adequate data to be included in the meta‐analysis. PCI score was reported as a continuous variable or condensed into categorical variables.

A PCI score greater than the following levels was predictive of reduced OS: PCI above 20 (unadjusted HR 2·90, 95 per cent c.i. 1·95 to 4·31, *I* ^2^ = 0 per cent; adjusted HR 5·04, 2·74 to 9·29, *I* ^2^ = 0 per cent); PCI above 15 (unadjusted HR 3·12, 2·02 to 4·80, *I* ^2^ = 0 per cent; adjusted HR 2·09, 1·39 to 3·15, *I* ^2^ = 0 per cent). PCI as a continuous variable was predictive of reduced OS: pooled unadjusted HR 1·07 (1·02 to 1·12) per increase of one PCI point (*I* ^2^ = 46·1 per cent); pooled adjusted HR 1·07 (1·04 to 1·11) per PCI point increase (*I* ^2^ = 67·1 per cent). Lower PCI levels were not predictive of OS: PCI above 10 (unadjusted pooled HR 2·57, 0·68 to 9·67; *I* ^2^ = 90 per cent); PCI above 6, adjusted pooled HR 3·28 (0·77 to 13·94; *I* ^2^ = 92 per cent) (*Fig*. 2; *Fig*. S1, supporting information).

### Treatment factors

#### *Previous surgical score*

Three studies^11^, ^34^, ^42^ reported on the influence of the previous surgical score (PSS) on OS. The pooled unadjusted HR for a PSS of at least 2 was 1·23 (95 per cent c.i. 0·78 to 1·95) (*I* ^2^ = 0 per cent) (*Fig*. 2; *Fig*. S1, supporting information). These data provide no evidence that PSS is a useful predictor of OS.

#### *Postoperative morbidity*

Three studies^40^, ^43^, ^50^ reported on the association between postoperative morbidity and OS. The pooled unadjusted HR for a Clavien–Dindo complication of grade III or above was 1·56 (95 per cent c.i. 0·74 to 3·31) (*I* ^2^ = 48·7 per cent) (*Fig*. 2; *Fig*. S1, supporting information). These data provide no evidence that postoperative morbidity is a useful predictor of OS.

#### *Neoadjuvant and adjuvant chemotherapy*

Neoadjuvant and adjuvant treatment of the primary tumour was reported poorly (*Table* S6, supporting information). No study reported response to treatment, or whether it was completed as planned.

Two studies^11^, ^43^ reported on the effect of neoadjuvant chemotherapy before CRS + HIPEC on OS. The pooled unadjusted HR for the use of neoadjuvant chemotherapy was 1·00 (95 per cent c.i. 0·63 to 1·58) (*I* ^2^ = 0 per cent) (*Fig*. 2; *Fig*. S1, supporting information). These data provide no evidence that neoadjuvant chemotherapy is a useful predictor of OS.

#### *Adjuvant chemotherapy*

Four studies^11^, ^35^, ^43^, ^50^ reported on the effect of adjuvant chemotherapy after CRS + HIPEC on OS. The pooled unadjusted HR for adjuvant chemotherapy was 0·60 (95 per cent c.i. 0·29 to 1·21) (*I* ^2^ = 68·1 per cent) (*Fig*. 2; *Fig*. S1, supporting information), which suggests there is no evidence that use of adjuvant chemotherapy is predictive of OS.

#### *Completeness of cytoreduction*

Eight studies^11^, ^32^, ^35^, ^40^, ^42^, ^43^, ^51^, ^55^ reported on the effect of the completeness of cytoreduction on OS; seven presented adequate data to be included in the meta‐analysis. A CC score above zero was predictive of a reduction in OS (unadjusted HR 1·75, 95 per cent c.i. 1·18 to 2·59) (*I* ^2^ = 79·5 per cent), as was a CC score of 2 or more (HR 1·61, 1·31 to 1·97) (*I* ^2^ = 0 per cent) (*Fig*. 2; *Fig*. S1, supporting information).

## Discussion

This systematic review and meta‐analysis examined the effect of prognostic factors on OS following CRS + HIPEC for CPM. Emergency presentation with obstruction or perforation of the primary tumour as well as the extent of CPM and the completeness of resection, as described by the PCI and the CC score respectively, were the only significant prognostic factors.

An emergency presentation of the primary tumour with obstruction or perforation was predictive of reduced OS (for both synchronous and metachronous CPM). A number of factors may contribute to this. In the primary setting, colorectal cancers presenting with obstruction or perforation are associated with decreased cancer‐specific survival and increased postoperative mortality^56^. Obstructed or perforated colorectal cancer is, by definition, advanced in stage and increases the risk of metastasis; additionally, emergency presentation limits the possibility of neoadjuvant treatment and may delay adjuvant treatment owing to postoperative morbidity. The extent of peritoneal metastasis as described by the PCI was predictive of reduced OS as a continuous variable and when the PCI score was 12 or above. A complete cytoreduction (CC0) was predictive of improved OS. Improving patient selection is therefore reliant on the ability to predict accurately the extent of peritoneal metastasis and the ability to resect it completely. Specialist radiologists have demonstrated good concordance between radiological and surgical PCI estimations, particularly when combining modalities; however, these tend to be most accurate in patients with high PCI scores^57^, ^58^. In some centres this is used in combination with diagnostic laparoscopy before CRS + HIPEC. This is feasible in the majority of patients and may help to reduce the laparotomy rate in patients for whom CRS + HIPEC may not be possible^59^.

Included patients were relatively young at 54 (range 45–69) years compared with the incident age of colorectal cancer (80–90 years)^60^ In addition, performance status was not reported by the majority of studies in the review, and limited to an ECOG score of less than 2 by a further nine. Within these limits, no other patient or tumour factor was predictive of OS.

Details of neoadjuvant and adjuvant treatments were reported poorly in the included studies. Within these limits, the use of neoadjuvant or adjuvant chemotherapy was not predictive of OS after CRS + HIPEC. One meta‐analysis, by Kwakman and colleagues^61^ from 2016, examined the effect of clinicopathological variables only on OS following CRS + HIPEC. Significant prognostic factors identified in the present review (adverse features of the primary tumour, PCI, CC score) were not comparable with those from Kwakman *et al*.^61^ as they were not examined. In contrast to the present study, Kwakman and co‐workers^61^ found performance status, the presence of lymph node or hepatic metastasis, tumour differentiation, signet ring histology, a rectal primary and the use of neoadjuvant chemotherapy to be predictive prognostic factors. A number of differences may explain this variation in findings: the exclusion of 73 papers described as unavailable in full text may introduce a potential selection bias^61^. In addition, a number of studies included by Kwakman and colleagues were excluded in the present study for the following reasons: presentation of inadequate data to estimate the HR accurately^13^, ^52^, ^54^, ^62^ the inclusion of mixed primary tumours^48^, ^63^, ^64^, and the inclusion of patients having repeat CRS + HIPEC procedures. Finally, the meta‐analysis^61^ combined all studies regardless of the presentation of unadjusted or adjusted HR.

The strengths of the present study include the comprehensive and systematic literature search including 3128 patients with CPM undergoing CRS + HIPEC, all potential prognostic factors were included, and a consistent association was found between predictive prognostic factors across different studies. The low heterogeneity associated with these factors adds to the strength and generalizability of the findings. The application of strict inclusion criteria limits the potential impact of factors such as mixed primary tumour origin. The present analysis takes into account the adjustment factors used in primary studies to ensure meta‐analysis of time to event data was performed only when data were comparable.

A number of limitations must, however, be acknowledged. This review is limited by the quality of primary studies and the heterogeneity of the population. Prognostic factor systematic reviews, by their nature, represent one of the most difficult categories due to their retrospective and observational nature. As CRS + HIPEC is performed at tertiary centres, data concerning the primary tumour and its treatment may not have been captured or reported fully. Additionally, the statistical analysis and presentation of data necessary for accurate meta‐analysis varied widely across primary studies, which may introduce a degree of inclusion bias.

There was a lack of molecular and genetic data concerning patients with CPM undergoing CRS + HIPEC in comparison with studies of primary colorectal cancer, and this is an area for future research. Discordance between primary genetic mutations and those in peritoneal metastases may identify novel therapeutic targets and prognostic markers in this metastatic group. Recent research by Schneider and colleagues^65^ found that *RAS*/*RAF* mutations impair survival after CRS/HIPEC, although this is the only study that has considered this prognostic factor and validation is required. Further large‐scale research is needed to account for these factors; this would require collaboration between CRS + HIPEC centres, standardization of the analysis and presentation of prognostic factor time to event data.

## Disclosure

The authors declare no conflict of interest.

## Supporting information


**Appendix S1**. Example search strategy: Medline
**Fig. S1**. Forest and funnel plots for each prognostic factor
**Table S1.** Patient demographics
**Table S2**. Adjustment factors used in multivariable analysis
**Table S3.** Risk‐of‐bias assessment results for each study using the Quality in Prognostic Studies (QUIPS) tool
**Table S4**. Outcomes
**Table S5**. Tumour factors
**Table S6**. Treatment factors
