# Supplementary material for: Meta‐analysis of prognostic factors for patients with colorectal peritoneal metastasis undergoing cytoreductive surgery and heated intraperitoneal chemotherapy
Source: BJS Open. 2019 Jun 27;3(5):585–94. doi: 10.1002/bjs5.50179 (PMC6773657; doi:10.1002/bjs5.50179)
Supplement: Supplementary file 1 — Appendix S1. Example search strategy: Medline Fig. S1. Forest and funnel plots for each prognostic factor Table S1. Patient demographics Table S2. Adjustment factors used in multivariable analysis Table S3. Risk‐of‐bias assessment results for each study using the Quality in Prognostic Studies (QUIPS) tool Table S4. Outcomes Table S5. Tumour factors Table S6. Treatment factors [file BJS5-3-585-s001.docx]

**BJS5_50179**

**Meta-analysis of prognostic factors for patients with colorectal peritoneal metastasis undergoing cytoreductive surgery and heated intraperitoneal chemotherapy**

**S. Hallam, R. Tyler, M. Price, A. Beggs and H. Youssef**

**Appendix S1 Example search strategy: Medline**

Search Strategy:

--------------------------------------------------------------------------------

1 exp Colorectal Neoplasms/

2 ((colon* or colorectal) adj2 (cancer or carcinoma or tumo$r or malignan*)).ti,ab.

3 exp Peritoneal Neoplasms/

4 ((peritoneal or peritoneum) adj2 (cancer or carcinoma* or malignan$ or spread* or neoplasm*)).ti,ab.

5 1 or 2

6 3 or 4

7 cytoreductive surgery.ti,ab.

8 ((intraperitoneal or intra-peritoneal) adj2 chemotherapy).ti,ab.

9 CRS.ti,ab.

10 HIPEC.ti,ab.

11 7 or 8 or 9 or 10

12 5 and 6

13 11 and 12

**Fig. S1 Forest and funnel plots for each prognostic factor**

**Table 1: Adjustment factors used in multi-variate analysis**

| **Study ID** | **Adjustment factors, multi-variate analysis** | | | | | | | | | | | | | | | | | | | |
| --- | --- | --- | --- | --- | --- | --- | --- | --- | --- | --- | --- | --- | --- | --- | --- | --- | --- | --- | --- | --- |
|  | **Age** | **Gender** | **Colon Rectum** | **ECOG** | **Lymph node** | **Adverse features** | **Hepatic metastasis** | **Signet ring Mucinous** | **Timing PM** | **PCI** | **CC score** | **GI anastomosis** | **MVR** | **R Status** | **Neo-adjuvant** | **Adjuvant** | **HIPEC EPIC SPIC** | **Morbidity** | **PSS** | **Adjustment factors in one paper only** |
| **Baratti**  **(2014)** |  |  |  | X |  |  | X |  | X | X | X |  |  |  |  |  |  | X |  |  |
| **Benziri**  **(2012)** |  |  |  |  |  |  |  |  |  | X | X |  |  |  |  |  |  |  |  | Small bowel. area involved |
| **Cashin**  **(2012)** |  | X |  |  |  |  |  |  |  | X |  |  |  |  | X | X | X |  | X | WBC count / Platelet count |
| **Da Silva**  **(2006)** | X |  |  |  | X | X |  |  |  | X |  |  |  |  |  |  |  |  |  |  |
| **Duraj**  **(2013)** | X | X | X |  | X |  | X |  |  | X |  | X |  | X |  |  | X |  | X | Tumour differentiation  Peritonectomy |
| **Elias**  **(2010)** |  |  |  |  | X |  |  |  |  | X | X |  |  |  |  | X |  |  |  |  |
| **Elias**  **(2013)** | X | X | X |  |  |  | X |  |  | X |  |  |  |  |  |  |  |  |  | Ovarian metastasis  Cardiphrenic LN |
| **Elias**  **(2014)** | X | X |  |  | X |  | X |  |  | X |  |  |  |  |  |  |  |  |  | Small bowel PCI |
| **Faron**  **(2016)** |  |  |  |  | X |  |  |  |  | X |  |  |  |  |  |  |  |  |  | Other metastasis |
| **Franko**  **(2008)** |  |  |  |  |  |  |  |  |  |  |  | X | X |  |  |  |  |  |  | Blood loss |
| **Froysnes**  **(2016)** | X | X | X |  | X |  |  |  |  | X |  |  |  |  |  |  |  |  |  | Disease free interval |
| **Huang**  **(2014)** |  |  |  |  |  |  |  |  |  | X | X |  |  |  |  |  |  |  |  |  |
| **Huang**  **(2016)** | X |  |  |  | X |  |  | X |  | X |  |  |  |  |  |  |  |  |  |  |
| **Ihemalndu**  **(2017)** | X | X |  |  |  |  |  | X |  |  | X |  |  |  |  |  |  |  | x | Tumour markers  Chemotherapy type  Surgery duration  Disease recurrence |
| **Lorimer**  **(2017)** |  |  | X |  | X |  | X | X |  | X | X | X | X |  |  |  |  | X |  |  |
| **Maggiori**  **(2013)** | X | X | X |  | X |  | X |  |  | X |  |  |  |  |  | X | X |  |  |  |
| **Ng**  **(2016)** |  |  |  |  |  |  |  |  |  | X |  |  |  |  |  |  |  |  | X |  |
| **Shen**  **(2004)** | X | X |  | X | X | X | X |  |  |  |  | X |  | X |  |  |  |  |  | Race  Ascites |
| **Sluiter**  **(2016)** | X |  |  |  | X |  |  |  |  | X |  |  |  |  |  |  |  |  |  | VEGF expression  VCAN expression |
| **Simkins,**  **Razenberg**  **(2016)** |  |  |  |  |  |  |  | X |  |  |  |  |  |  |  |  |  |  |  |  |
| **Simkens,**  **Van Oudheusden**  **(2016)** |  |  | X |  |  |  |  |  |  |  |  |  |  |  |  |  |  |  |  |  |
| **Ung**  **(2013)** | X |  |  |  | X |  |  |  |  | X |  |  |  |  |  |  |  |  |  |  |
| **Winer**  **(2014)** | X | X |  |  |  |  |  |  |  |  | X |  |  |  |  |  |  | X |  | ASA  Albumin |

**Table 1: Adjustment factors used in multi-variate analysis** (ECOG Eastern Cooperative Oncology Group, PM peritoneal metastasis, PCI Peritoneal carcinomatosis index, CC score completeness of cytoreduction, GI gastrointestinal, MVR multivisceral resection, R status resection status, HIPEC heated intra-peritoneal chemotherpay, EPIC Early post-operative chemotherapy, SPIC Sequential postoperative intraperitoneal chemotherapy ASA American society of anaesthesiologists score VEGF, Vascular endothelial growth factor, VCAN Versican, , LN lymph node, WBC white blood cell)

Table 2: Patient demographics

| **Author**  **(year)** | **Country of origin** | **Study design** | **Number patients** | **Age, years (range)** | **Gender**  **(M:F)** | **Follow up**  **(months, median (range)** | **Co- morbidity (range)** | **Performance status** |
| --- | --- | --- | --- | --- | --- | --- | --- | --- |
| **Baratti**  **(2014)** | Italy | RC | 101 | 60 (29-79) | 40:61 | 44.9 (24.1-65.7 95%CI) | Charlston score 4 (2-7) | ECOG 0 54  ECOG 1 42  ECOG 2 5 |
| **Beniziri (2012)** | France | RC | 49 | 52.7 x̅ +/-SD 11 | 19:30 | 27 x̅ +/-8 | NS | ECOG <2 |
| **Cashin**  **(2012)** | Sweden | RC | 126 | 55 x̅ (14 – 79) | 76:75 | 49 (0.5-200) | NS | WHO <2 |
| **Da Silva (2006)** | USA | RC | 70 | 45.5 x̅ (18-71) | 27:43 | 46.5 x̅ (6-241) | NS | NS |
| **Duraj**  **(2013)** | Sweden | PC | 11 LM+PM  22 PM | 60 LM+PM  57 PM | 2:9 LM+PM  7:15 PM | 57 LM+PM, 45 PM | NS | NS |
| **Elias**  **(2010)** | International centre (n23) | RC | 523 | 54 (16-88) | 227:296 | 45 (23-79 IQR) | NS | WHO0 n=387 (76)  WHO1 n=102 (20)  WHO2 n=20 (4) |
| **Elias**  **(2013)** | France | RC | 114 | 50 +/-SD 10 | 49:65 | 46.8 (0-140.4) | NS | NS |
| **Elias**  **(2014)** | France | PC | 139 | SB 49.1  NSB 49.7 | SB 37:63  NSB 14:25 | 62.4 (55.6-77.6) | NS | NS |
| **Faron**  **(2016)** | France | RC | 173 | 48.9 (20-74) | 71:102 | 48 (41.2-56.3) | NS | NS |
| **Franko**  **(2008)** | USA | RC | 65 | 51.6+/- SD 13.2 | 18:47 | NS | NS | NS |
| **Froysnes (2016)** | Norway | RC | 119 | 58 (22-77) | 42:77 | 45 | NS | All ECOG 0-1 |
| **Huang**  **(2014)** | China | PC | 60 | < 60 n46  > 60 n14 | 26:34 | NS | NS | NS |
| **Huang**  **(2016)** | Australia | RC | 168 | 534 x̅ SD 14.1 | 51:104 | 20.1 (0.2-112) | NS | NS |
| **Ihemelandu (2017)** | USA | RC | 318 | 50.6 x̅ (18-86) | 171:147 | 15 (3-264) | NS | NS |
| **Lorimer (2017)** | France | RC | 22 PM+LM  36 PM | 60 (21-77) median NS 0.82 | 29:29 | 29.2 (5-125) | NS | ECOG <2 all |
| **Maggiori (2013)** | France | PC | 37 PM+LM  61 PM | PM+LM 49 (27-67)  PM: 50 (25-63) | PM+LM: 11:26  PM: 16:45 | 36+/-28 | NS | ECOG <2 all |
| **Ng**  **(2016)** | Singapore | RC | 50 | 50 (14 – 71) | 18:32 | 13.3 (0.8 – 87.1) | n=31 yes | ECOG 0 n=41 (82)  ECOG 1 n=9 (18) |
| **Shen**  **(2004)** | USA | PC | 77 | 54 x̅ | 45:32 | 15 | NS | ECOG 0 n=18 (23)  ECOG 1 n=46 (60)  ECOG 2 n=7 (9)  ECOG 3 n=2 (3)  ECOG 4 n=4 (5) |
| **Simkins,**  **Razenberg**  **(2016)** | Netherlands | RC | 445 | 69.3+/-SD 11.6 AC  68.5+/-SD 12.1 MC  65.3+/-SD 14.2 SR | NS | NS | NS | NS |
| **Simkens,**  **Van Oudheusden (2016)** | Netherlands | PC | 58 colon  29 rectal | 59.4+/-SD 11.2  62.3 +/-SD 9.4 | 14:15 rectal  28:30 colon | 21.8 (3.2-71.3) rectal  21.3 (2.5-96.6) colon | NS | Colon:rectum  ASA 1 4:5  ASA 2 22:45  ASA 3 3:5 |
| **Sluiter (2016** | Netherlands | RC | 65 | 62 (31-78) | 25:40 | 21 (2-50) | NS | NS |
| **Teo (2015)** | Singapore | RC | 35 | 51 (14-71) | 11:24 | 24.7 (0.6-81.8) | NS | ECOG ≤1 n=35 (100) |
| **Ung (2013)** | Australia | RC | 125 | 58.9 (NS) | 50:75 | 23.3 (1-156) | NS | NS |
| **Winer (2014)** | USA | RC | 30 | 54.9 x̅ +/-SD 14.5 | 22:8 | 52 (IQR 1.04-11.5) | NS | NS |

**Table 2: Patient demographics** (NS not specified, RC retrospective cohort, PC prospective cohort, LM liver metastasis, PM peritoneal metastasis, AC adenocarcinoma, MC mucinous, SR signet ring, ECOG Eastern Cooperative Oncology Group performance status score, ASA American society of anaesthesiologists score, SB small bowel, NSB not small bowel, NS not specified, x̅ mean, IQR inter-quartile range, CI confidence interval, SD standard deviation, n number followed by value in parenthesis, percentag

**Table 3: Risk of bias assessment results using the Quality in Prognostic Studies (QUIPS) tool**

| **Study ID** | **Study participation** | **Study attrition** | **Prognostic factor measurement** | **Outcome measurement** | **Study confounding** | **Statistical analysis and reporting** |
| --- | --- | --- | --- | --- | --- | --- |
| **Baratti (2014)** | Moderate | Moderate | Low | Low | Moderate | Low |
| **Benziri (2012)** | Low | Moderate | Low | Moderate | High | Low |
| **Cashin (2012)** | Low | Moderate | Low | Low | Moderate | Moderate |
| **Da Silva (2006)** | Moderate | High | High | Moderate | Moderate | Low |
| **Duraj (2013)** | Low | Moderate | Low | Low | Moderate | Low |
| **Elias (2010)** | Low | Moderate | Low | Moderate | Moderate | Low |
| **Elias (2013)** | Moderate | Low | High | Low | Moderate | Low |
| **Elias (2014)** | Low | Moderate | Low | Moderate | High | Low |
| **Faron (2016)** | Low | Moderate | Low | Low | Moderate | Moderate |
| **Franko (2008)** | Moderate | Moderate | Low | Moderate | Moderate | Low |
| **Froysnes (2016)** | Low | Moderate | Low | Low | Moderate | Low |
| **Huang (2014)** | Low | Low | Low | Low | Moderate | Low |
| **Huang (2016)** | Low | Moderate | Low | Moderate | Moderate | Low |
| **Ihemalndu (2017)** | Low | Moderate | Low | Moderate | Moderate | Low |
| **Lorimer (2017)** | Low | Moderate | Low | Low | Moderate | Low |
| **Maggiori (2013)** | Low | Low | Low | Low | Moderate | Low |
| **Ng (2016)** | High | High | Low | Moderate | Moderate | Low |
| **Shen (2004)** | Low | Moderate | Low | Moderate | Moderate | Low |
| **Sluiter (2016)** | Low | Moderate | Moderate | Moderate | High | Low |
| **Simkins,**  **Razenberg (2016)** | High | High | Moderate | Moderate | Moderate | Low |
| **Simkens,**  **Van Oudheusden (2016)** | Low | Moderate | Moderate | Moderate | Moderate | Low |
| **Teo (2015)** | Low | Low | Low | Low | Moderate | Low |
| **Ung (2013)** | Low | Moderate | Low | Moderate | Moderate | Low |
| **Winer (2014)** | Moderate | Moderate | Low | Mow | Moderate | Low |

**Table 3: Risk of bias assessment results for each study using the Quality in Prognostic Studies (QUIPS) tool.** *Each study is judged to have a low, moderate, high or none applicable risk of bias from each domain of the QUIPS tool*

**Table 4: Outcomes**

| **Author (year)** | **Deaths** | **Median OS (95% CI)** | **5 year OS (95% CI)** |
| --- | --- | --- | --- |
| **Baratti**  **(2014)** | 37 (31 disease related) | 32 (16.2-55.9) | 11.7% (9.5-24.5) |
| **Beniziri (2012)** | NS | 51 (NS) | NS |
| **Cashin (2012)** | NS | 34 (2-77) CRS+HIPEC  25 (2-188) CRS+SPIC, p0.047 | 40% CRS+HIPEC  18% CRS+SPIC, p0.047 |
| **Da Silva (2006)** | NS | 33 (NS) | 32% (NS) |
| **Duraj (2013)** | NS | 15 (6-46) PM+LM  34 (19-37) PM, p=0.2 | NS |
| **Elias (2010)** | NS | 30.1 (NS) | 27% (21-33%) |
| **Elias (2013)** | 47/114 | NS | NS |
| **Elias (2014)** |  | SB 30.9 x̃ (0.23-113)  NSB 45.5 x̃ (0.79-144) | 39% (NS) |
| **Faron (2016)** | NS | 41 (32-50) | 42% (NS) |
| **Franko (2008)** | NS | 15.3 (NS)  20.2 R0/1  MVR 32.8 (NS) / Controls 20 p0.787 | NS |
| **Froysnes (2016)** | NS | 47 (42-52) | NS |
| **Huang (2014)** | NS | 16 (12.2-19.8) | 22% |
| **Huang (2016)** | NS | 42.1 (33.7-50.4) | 34% (NS) |
| **Ihemelandu (2017)** | NS | 21.5 (NS) | 25% (NS) |
| **Lorimer (2017)** | NS | PM 25.2 (14.8-82.6)  PM+LM 36.1 (19.6-113.7), p0.3 | PM: 40.7% (23.4-77.3)  PM+LM: 42.1% (19.7-63) |
| **Maggiori (2013)** | NS | NS | PM+LM: 26% (18-35)  PM: 43% (35-51) |
| **Ng (2016)** | 18/50 (36) | x̃ 28.8 (18.3-39.1) | NS |
| **Shen (2004)** | NS | 16 (10 – 26) | 17% (NS) |
| **Simkins,**  **Razenberg**  **(2016)** | NS | 32.8 (27.8-37.8) | NS |
| **Simkens,**  **Van Oudheusden**  **(2016)** | NS | 26 (22.2-29.9) rectal  35.1 (22.8-47.3) colon | 32% (NS) rectal  24% (NS) colon |
| **Sluiter (2016)** | NS | 34.4 (29.4-39.2) | NS |
| **Teo (2015)** | 13/35 (37) | 27.1 (15.3-39.1) | 19.1 (NS) |
| **Ung (2013)** | NS | 37.1 (NS) colon  29.6 (NS) rectum | 33% (NS) colon  20% (NS) rectum |
| **Winer**  **(2014)** | 25/30 (83) | 12.2 (7.5-17.2) | NS |

**Table 4: Outcomes: (**NS not specified, NK not known, x̃ median with (range), PM: Peritoneal metastasis, LM liver metastasis, SB small bowel, NSB none small bowel,CRS cytoreductive surgery, HIPEC heated intra-peritoneal chemotherapy, EPIC Early post-operative intraperitoneal chemotherapy, SPIC sequential post-operative intraperitoneal chemotherapy)

**Table 5: Tumour factors**

| **Author**  **(year)** | **Synch: Metach** | **DFI (month)** | **Colonic: Rectal** | **T stage** | **N stage** | **Distant metastasis** | **Differentiation**  **Well:Mod:Poor** | **Histological type** | **PCI (range)** | **CC score**  **0:1:2:3** |
| --- | --- | --- | --- | --- | --- | --- | --- | --- | --- | --- |
| **Baratti**  **(2014)** | 48:54 | NS | 92:7  RS 1  NS 1 | NS | NS | LM n=8 | 8:68:25 | NS | 10 (1-39) | 87:12:2:0 |
| **Beniziri**  **(2012)** | NS | 20 x̅ +/- SD 9 | 8:41 | NS | N+ n=22  N- n=27 | NS | 6:35:8 | NS | 10 x̅ +/-SD 6.2 | 37:12:0:0 |
| **Cashin**  **(2012)** | 102:48 | NS | 135:15 | NS | NS | Excluded | NS | MC n=89  SR n=18 | 1-10 n=49  11-20 n=45  21-39 n=56 | 97:0:0:0  0:≥54 |
| **Da Silva**  **(2006)** | NS | <12 n35  >12 n27 | 64:6 | NS | N0 n=14  N1/2 n=53  NS n=3 | NS | 11:50:9 | MC n=36  SR n=5 | <20 n=60  >20 n=10 | 0/1 n=70 |
| **Duraj**  **(2013)** | 8:3 PM+LM  15:7 PM | NS | 9:2 PM+HM  18:4 PM | NS | N+ n=8, N0 n=3 PM+HM,  N+ n=14,N0 n=6, NS2 PM | LM | 0:10:0 PM+HM,  2:13:7 PM | MC n=5/11, n=11/22 | NS | R*  R1 n=10  R2 n=1 PM+HM R1 n=20  R2 n=2 PM |
| **Elias**  **(2010)** | 161:300 | NS | 433:36  RS n=8  NS n=73 | NS | LN+ 325  N0 n=158 | LM n=77 | NS | NS | 1-6 n=181 (37)  7-12 n=132 (28)  13-19 n=96 (21)  >19 n=69 (14) | 439:53:22:0 |
| **Elias**  **(2013)** | NS | NS | 94:23 | NS | NS | LM n=24  Ovarian n=46 | NS | NS | 9.2 x̅ (0-27) | 0/1 n=114 |
| **Elias**  **(2014)** | NS | NS | NS | NS | NS | LM  SB n=16, NSB n=11 | SB 50:0:18  NSB 19:0:9 | SB MC n=28, SR n=10  NSB MC n=10, SR n=3 | SB median 12 (3-36)  NSB 4 (2-11)  p<0.0001 | 0/1 n=139 |
| **Faron**  **(2016)** | NS | NS | 135:38 | NS | NS | LM n=52 | NS | NS | 10.2 x̅ +/-6.8 | 0/1 n=173 |
| **Franko**  **(2008)** | NS | NS | NS | NS | NS | NS | NS | NS | NS | NS |
| **Froysnes**  **(2016)** | 73:46 | 15 median (7-67) | 109:10 | T1 n=1  T3 n=54  T4 n=63  NS n=1 | N0 n=34  N1 n=41  N2 n=42  N2 n=2 | Excluded | 8:67:33  NS n=11 | SR n=14  MC n=33 | Median 9 (0-28)  0-10 n=74  11-20 n=35  >20 n=10 | 113:5:0:0  NS n=1 |
| **Huang**  **(2014)** | 24:36 | NS | 35:25 | NS | NS | NS | AC, well / mod diff. n=26  AC, poor diff. / MC / SR n=34 | | <20 n=28  > 20 n=32  Median 21 (1-39) | 17:15: ≥ 28 |
| **Huang**  **(2016)** | 168 metach (100) | NS | NS | NS | n=102 RPLN | Excluded | 3:97:27  Benign n=22 | SR n=11 | 9.5 x̅ +/- SD 6.6 | 0/1 n=168 |
| **Ihemelandu (2017)** | NS | NS | NS | NS | NS | NS | NS | AC n=150  SR n=11 | 15.2 x̅ +/-SD 11.2 | ≤1 188:37:80 |
| **Lorimer**  **(2017)** | 58 (100) synch | NA | 54:4 | T>2 n=55 | N+ n=43 | LM n=22 | NS | MC n=16/58 | 13 median (1-39) | 44:9:5:0 |
| **Maggiori**  **(2013)** | 98 (100) synch | NA | 50:11 PM  32:5 PM+LM | PM: T/2 n=1, T3/4 n=60  PM+LM T3/4 n=37 | PM: N+ n=54, N- n=7  PM+LM N+ n=31, N- n=6 | LM, n=37 | NS | NS | PM: 9 (2-26)  PM+LM: 11 (1-26) | 0/1 n=98 |
| **Ng**  **(2016)** | NS | NS | 49:1 | NS | NS | NS | 0:27:13 | MC 26%  SR 2% | 10 median (1-27) | 49:1:0:0 |
| **Shen**  **(2004)** | 21:56 | 14 (3-85) | 74:3 | NS | NS | LM n=10  None n=65  NS n=2 | NS | MC n=43 (56)  SR n=7 (9)  NS n=27 (35) | NS | R0/1 n=37  >R1 n=40 |
| **Simkins,**  **Razenberg**  **(2016)** | 445 (100)  Synch | NA | NS | NS | NS | NS | NS | AC n246 (55)  MC n156 (35)  SR n43 (10) | NS | NS |
| **Simkens,**  **Van Oudheusden (2016)** | 19:10 rectal  31:27 colon | NS | 58:29 | Rectal:  colon  ≤T3 19:38  ≥T4 9:20 | Rectal:colon  N0 7:17  N1 9:17  N2 12:24 | LM  Rectal n=3  Colon n=7 | Rectal:colon  Good-mod 19:34  Poor 2:8 | Rectal:  colon  MC 9:5  SC 6:3 | Rectum 9.3 +/- 4.7  Colon 9 +/- 5.1 | All R1 |
| **Sluiter**  **(2016)** | 35:30 | NS | 49:5  11 RS | T1 n=1  T2 n=2  T3 n=27  T4 n=35 | Positive n46  Negative n17  NS n2 | NS | 3:22:11  NS n=29 | AC n=39  MC n=22  SC n=4 | *SPCI  <2 n1  2-4 n=42  5 n=14  >5 n=8 | R1 n=37  R2a n=22  R2b n=6 |
| **Teo**  **(2015)** | NS | 15 (1.7-95.9) | NS  15 (43) Right  20 (57) Left | T1 n=1  T2 n=0  T3 n=9  T4 n=22 | N0 10  N1 10  N2 10 | Excluded | NS | AC n=23  MC n=12 | 12 (1-27) | 33:1:1:0 |
| **Ung**  **(2013)** | NS | NS | 109:16 | NS | N+ 84  N0 41 | LM n=51 (41) | 8:85:32 | MC n=56 (45) | 1-6 n=43  7-12 n=46  13-19 n=24  >20 n=12 | 120:5:0:0 |
| **Winer**  **(2014)** | 21:9 | NS | NS | NS | NS | NS | NS | SR n30 (100) | 11.5 (1-19) | 14:9:2:2  0  NS n=3 |

**Table 5: Tumour factors:** (Synch synchronous, metach metachronous, RS rectosigmoid, RPLN retroperitoneal lymph node, DFI disease free interval, NS not specified, NA not applicable, LM liver metastasis, PM peritoneal metastasis, AC adenocarcinoma, MC mucinous, SR signet ring, PCI peritoneal carcinomatosis index, sPCI short peritoneal carcinomatosis index, LM liver metastasis, CC completeness of cytoreduction score, R0-2 completeness of resection, CRS cytoreductive surgery, HIPEC heated intra-peritoneal chemotherapy, SB small bowel, NSB not small bowel , NS not specified, x̅ mean, CI confidence interval, SD standard deviation, n number followed by value in parenthesis, percentage))

**Table 6: Treatment factors**

| **Author**  **(year)** | **Neo-adjuvant therapy** | | | **CRS method** | **HIPEC** | | | | **EPIC / SPIC** | **Surgery for metastasis** | **Adjuvant therapy** | | |
| --- | --- | --- | --- | --- | --- | --- | --- | --- | --- | --- | --- | --- | --- |
|  | **No (%)** | **Type** | **Completed** |  | **Agent** | **Duration (min)** | | **Temp (C)** |  |  | **No (%)** | **Type** | **Completed** |
| **Baratti**  **(2014)** | 87/101 (86) | 5-FU/CAP n24,  5-FU + OXA n12,  5-FU + OXA + bevacizumab,  5FU/CAP + OXA n26  5FU +OXA + bevacizumab or Cetuximab n11,  5-FU based n6,  Other n3 | NS | Complete macroscopic resection | CIS 25ml/m^2^ + MMC 3.3mg/m^2^ | 60 | 42.5 | | NA | 8 liver resection  4 RFA liver | 70/101 (69) | NS | NS |
| **Beniziri (2012)** | 37/49 (72.5) | NS | NS | NS | MMC  12.5mg/m^2^ M  10mg/m^2^ F | 90 | 42 | | NA | NS | NS | NS | NS |
| **Cashin**  **(2012)** | 47/126 | NS | NS | Sugarbaker | HIPEC n=67  MMC 30mg/m^2^ (n2)  OXA 460mg/m^2^ /+ IV 5-FU 400mg/mg + calcium folinate 60mg/m^2^ (n44)  OXA 360mg/m^2^ + IRI 360mg/m^2^ + IV 5-FU 450-500mg/m^2^ + calcium folinate 60mg/m^2^  (n=23) | 90  30  30 | 41 - 42 | | SPIC*  5FU 500-600mg/m2 + IV LV 60mg/m2 daily 7 days  8x cycles in 6 months | NA | 27/126 | NS | NS |
| **Da Silva (2006)** | NS | NS | NS | Sugarbaker | n=34 (after 1997)  MMCC 10mg/m^2^ F/12mg/m^2^ M  + 5x days 5-FU 650mg/m^2^ | 90 | 41-42C | | n=36 EPIC alone  MMC 10mg/m2 F/12mg/m2 M  + IV 5-FU 650mg/m2 23 hrs + 6x days EPIC 5-FU  Adjuvant 6/12 (1x week) as above | NS | NS | NS | NS |
| **Duraj**  **(2013)** | Y:N  10:1 PM/HM  13:9  PM | NS | NS | Complete macroscopic resection | n=13 OXA 460mg/m^2^  IP + 5-FU 400mg/m2/LV 60mg/m^2^ IV  n= 7 OXA 360mg/m^2^ + IRI 360mg/m^2^  + IV 5-FU 400mg/m^2^/LV 60mg/m^2^ | 30  30 | 41-42 | | n=8 + EPIC  5-FU 550mg/m2 IP + LV 60mg/m2  n12 +SPIC 5-FU 500-600mg/m2 + LV 60mg/m2 6 days, 8x cycles over 6 months | LM  n=2 hemihepatectomy  n12 Segmental | 4/11 PM/HM  7/22 PM | NS | NS |
| **Elias**  **(2010)** | 370 (70) | NS | NS | Complete macroscopic resection | MMC 30-50mg/m^2^ +/- CIS 50-100mg/m^2^  OR  OXA 360-460mg/m^2^ +/- IRI 200mg/m^2^ + IV 5-FU + LV, | 60-120  30 | 41  43 | | MMC 10mg/m2 D1  5-FU 600mg/m2 days 2-5 | Synchronous liver resection n77 | 232 (47%) if objective response to neoadjuvant OR poor prognostic factors CC1-2, LN+, liver mets | NS | NS |

|  |  |  |  |  |  |  |  |  |  |  |  |  |
| --- | --- | --- | --- | --- | --- | --- | --- | --- | --- | --- | --- | --- |
| **Elias**  **(2013)** | 114 (100) | NS | NS | Complete macroscopic resection | OXA 460mg/m^2^  OR  OXA 300mg/m^2^ + IRI 200mg/m^2^  +IV 5-FU 400mg/m^2^ / LV 20mg/m^2^ | 30  30 | 43  43 | NA | Synchronous liver n=24 | 84% | NS | NS |
| **Elias**  **(2014)** | 139 (100) | NS | NS | Complete macroscopic resection | OXA 460-mg/m^2^  OR  OXA 300mg/m^2^ + IRI 200mg/m^2^  IV 5-FU 400mg/m^2^ + LV 20mg/m^2^ | 30  30 | 42  42 | NA | Synchronous liver n=37 | 84% | NS | NS |
| **Faron**  **(2016)** | 2 months | NS | NS | Complete macroscopic resection | 300mg/m^2^ OXA + 200mg/m^2^ IRI  IV 400mg/m^2^ 5FU + LV 20mg/m^2^ | 30 | 42-43 | NA | 30.2% liver resection | 6 months total including neo-adjuvant | NS | NS |
| **Franko**  **(2008)** | 64/65 | Oxalaplatin n=37, Irinotecan n16, Bevacizumab n21 | NS | Complete macroscopic resection | MMC 40mg | 100 | NS | NA | NS | NS | NS | NS |
| **Froysnes (2016)** | 81/119 | NS | NS | Sugarbaker | MMC 35mg/m^2^ | 90 | 41.4 | N=16 additional HIPEC usually within 1x week CRS | NA | Not routinely given | NA | NA |
| **Huang**  **(2014)** | median cycles 8 (2-18), median, range  <6 n=15  >6 n=45 | NS | NS | Complete macroscopic resection | 120mg CIS +30mg MMC | 90 | 43+/-0.5C | Once ‘postoperative physical condition recovered’  DOC 75mg/m2 + CBDCA day 1 every 3 weeks  With adjuvant or alternate cycles  Median 4 cycles (1-10) | NS | FOLFOX or FOLFIRI | NA | NA |
| **Huang**  **(2016)** | NS | NS | NS | Sugarbaker | OXA 350mg/m2  OR MMC 12.5mg/m2 | 30 | 42 | NS | NA | NS | NS | NS |
|  |  |  |  |  | HIPEC n161, EPIC n=7, HIPEC+EPIC n48 | | | |  |  |  |  |
| **Ihemelandu (2017)** | Recommend 3-4 cycles | 5FU or Capecitabine + Oxalaplatin +/- bevacizumab | NS | Complete macroscopic resection | <1995 – MMC 15mg / m^2^  >1995 – MMC 15mg / m^2^  >2010 MMC + DOX 15mg/m^2^ | 90 | Cold  42  42 | 5-FU 400 (F), 600 (M) for 5 days post op for C1 / 2 patients  After 1995 + LV 20mg/m2  (Not if ‘massive cytoreduction’, or long term systemic chemo before CRS | NS | NS | NS | NS |

| **Lorimer (2017)** | 2-3 months with response | NS | NS | Complete macroscopic resection | 2002 – OXA 460mg/m^2^  <2003 – MMC 30mg/m^2^ + CIS 100mg/m2 | 30  30 | 42-45  42-45 | NA | Minor hepatectomy n=17  Major hepatectomy n=5 | PM 20 (90.9)  PM+LM 30 (80.3) | NS | NS |
| --- | --- | --- | --- | --- | --- | --- | --- | --- | --- | --- | --- | --- |
| **Maggiori (2013)** | 2-3/12 pre op – no progression on tx | NS | NS | Complete macroscopic resection | 460mg/m2 OXA  +IV 5-FU 400mg/m2 + LV 20mg/m2 | 30 | 43-45 | MMC+5FU | Minor hepatectomy n=25  Major hepatectomy n12  +/-RFA n7 | PM n=48  PM+LM n30 | NS | NS |
|  |  |  |  |  | PM: n=49 HIPEC alone, n=11 EPIC alone, n=1 HIPEC+EPIC  PM+LM: n=16 HIPEC alone, n=3 HIPEC+EPIC, n=18 EPIC alone | | | |  |  |  |  |
| **Ng (2016)** | NS | NS | NS | Complete macroscopic resection | MMC  OR  OXA + LV + 5-FU  (dose NS) | 60-90 | 39-43 | NA | NS | n=11 yes  n=26 no  n13 NK | NS | NS |
| **Shen**  **(2004)** | 58/77 (75) | NS | NS | Complete macroscopic resection | MMC C 40mg  *dose reduction 30mg – elderly, extensive prior chemo, poor performance status extensive peritonectomy | 120  60-90 | 39.5-43 | NA | n=10 synchronous liver | ‘some’ | NS | NS |
| **Simkins,**  **Razenberg**  **(2016)** | NS | NS | NS | Sugarbaker | MMC C 35mg/m^2^ | 90 | 41-42 | NS | NS | NS | NS | NS |
| **Simkens,**  **Van Oudheusden (2016)** | 14/29 (48.3) rectal  7/58 (12.1) colon | NS | NS | Complete macroscopic resection | MMC C 25mg/m^2^  OXA 460mg/m^2^ + IV 4FU, LV 20mg/m^2^ | 90  30 | NS | NS | 5 pre CRS+HIPEC  4 synchronous  *1 not resected | n=21 (72) rectal  n=40 (69) colon | NS | NS |
| **Sluiter**  **(2016)** | 17/65 (26) | NS | NS | Sugarbaker | MMC C 35mg/m^2^ | 90 | 39-41 | NA | NS | 41/65 (63) | NA | NA |
| **Teo**  **(2015)** | NS | NS | NS | Sugarbaker | MMC dose NS | 60 | 42 | All + EPIC  5-FU 5 days, dose NS | NA (excluded) | ‘most’ | NA | NA |
| **Ung**  **(2013)** | 76/125 (61) | NS | NS | Sugarbaker | MMC 12.5mg/m^2^ | NS | 42 | 5-FU 5 days 650mg/m2 | 51 synchronous | 99/125 (79) | NS | NS |
|  |  |  |  |  | HIPEC n=58, EPIC n=20, HIPEC+EPICn=47 | | | |  |  |  |  |
| **Winer**  **(2014)** | 25/30 (83) | NS | NS | Complete macroscopic resection | MMC 40mg | 40 | 42 | NA | NS | NS | NS | NS |

**Table 6: Treatment factors** (MMC Mitomycin C, OXA Oxalaplatin, LV Leucovorin, 5-FU 5 Fluorouracil, CIS Cisplatin, DOX Doxorubicin, DOC docetaxel, CBDCA carboplatin, IRI Irinotecan, CAP Capecitabine, mg milligrams, m^2^ metres squared, Min minute, C centigrade, EPIC Early post-operative intraperitoneal chemotherapy, SPIC sequential post-operative intraperitoneal chemotherapy, CRS cytoreductive surgery, HIPEC heated intra-peritoneal chemotherapy, NA not applicable, NS not specified, deviation, n number followed by value in parenthesis, percentage)))
